# User-Reported Issues With Mental Health Apps: Machine-Assisted Topic Analysis of Social Media Posts

**DOI:** 10.2196/85575

**Published:** 2026-07-21

**Authors:** Jack Bolter, Trisevgeni Papakonstantinou, Paulina Bondaronek

**Affiliations:** 1 Department of Psychology, School of Social Sciences, University of Westminster, London, United Kingdom; 2 Department of Computer Science, University College London, London, United Kingdom; 3 Institute of Health Informatics, University College London, 222 Euston Road, London, NW1 2DA, United Kingdom, 44 07742966769

**Keywords:** health behavior, mobile apps, health promotion, mHealth, human-computer interaction, mental health apps, user experiences, digital mental health, machine learning, qualitative data, Twitter, application programming interface, API, mobile health

## Abstract

**Background:**

Mobile apps marketed to support mental health have become increasingly popular in recent years. Given their widespread use, it is important to identify issues that users experience while using such apps. Understanding these issues may provide insight into the safety and suitability of these apps for individuals seeking mental health support.

**Objective:**

Unlike existing research, where user experience issues have been identified through researchers’ direct analysis of apps, this study aimed to generate themes relating to user experience issues using comments from app users themselves. An additional aim was to evaluate a human-in-the-loop machine learning approach using structural topic modeling (STM) to analyze vast volumes of data gathered from X (formerly Twitter, developed by Twitter, Inc).

**Methods:**

Data relating to five of the most popular mental health apps were collected from the X API using R. A machine-assisted thematic analysis approach combined STM with human qualitative analysis to interpret user-generated posts. An unsupervised topic-modeling approach was tested using models with 5-40 topics and differing covariates (ultimately, a model without covariates was selected). Two researchers independently conducted thematic analysis to interpret and contextualize model outputs. A structural topic model with 10 topics, each comprising 20 X posts, was selected as most appropriate for generating insights.

**Results:**

Using R (developed by the R Core Team), 79,703 X posts were collected via the X API relating to five popular mental health apps. After negative sentiment filtering, 19,603 posts remained. Posts spanned March 2006 (the launch of X/formerly Twitter) to December 2022. Researchers collaboratively labeled the 10 topics to identify the primary user experience issue represented in each. Topic 3 was discarded due to low coherence and inconsistency in relation to app user experience, and Topic 5 was discarded because posts reflected app X account activity rather than user experience of the apps. The remaining eight topics were organized into four themes. The first theme, guidance shortfall, included difficulties following guided meditations, challenges selecting appropriate content from large libraries, and incompatibility between app use and home environments. The second theme, technical difficulties, involved subscription access issues and technical faults within apps. The third theme, heightened emotions related to app-affiliated celebrities, captured both over-excitement linked to celebrity involvement and anger directed toward specific celebrities. The final standalone theme, negative impacts of sleep self-monitoring, demonstrated users reporting that tracking sleep adversely affected sleep experience.

**Conclusions:**

The combination of STM and human qualitative analysis of X posts identified several user-experienced issues associated with popular mental health apps, often linked to negative outcomes. This study provides evidence that STM can be combined with qualitative methods to rapidly analyze large-scale social media data and generate insights into user experience of mass-reach digital health interventions.

## Introduction

In England, 1 in 4 people experience mental health issues annually, with 1 in 6 reporting common problems such as anxiety or depression on a weekly basis [1,2]. Depression is the leading global cause of disability [3], and severe mental illness can reduce life expectancy by up to 20 years due to preventable physical conditions [4]. People with poor mental health may feel down, irritable, hopeless, and exhibit behavior changes such as social withdrawal, self-harm, sleep loss, increased use of drugs and alcohol, and altered eating habits [5]. Only one-third of adults in England and Wales with common mental health conditions receive treatment [2,6], despite the World Health Organization noting globally low treatment rates [4]. Barriers to seeking help include limited knowledge about mental illness, unawareness of treatment options, and fear of stigma [7].

Given the low uptake of formal support, individuals often turn to digital alternatives. The mental health app market, valued at $5.2 billion in 2022, is projected to grow at 18% annually until 2030 [8]. Popular apps such as Calm (developed by Calm.com, Inc), Headspace (developed by Headspace, Inc), and Sleep Cycle (developed by Sleep Cycle AB) offer mindfulness, relaxation, and sleep-related features, with Calm alone exceeding 107 million downloads between 2018 and 2022 [9]. However, concerns remain regarding app effectiveness, as many apps lack empirical support, involve few health care professionals in development, and provide limited or inaccurate tracking of mental health metrics [10-15]. The unregulated nature of this market, coupled with the prioritization of venture capital-driven growth, may contribute to these shortcomings [16-18].

This study is situated within the broader landscape of mobile health (mHealth) apps, a heterogeneous category encompassing digital tools designed to support health and well-being through mobile devices [19]. Within this ecosystem, mental health apps represent a diverse subset that spans multiple functional domains, including mindfulness and meditation, sleep improvement, mood regulation, psychoeducation, and passive or active self-tracking [20]. The five apps analyzed in this study—Calm, Headspace, BetterSleep (developed by Ipnos Software Inc), Sleep Cycle, and Waking Up (developed by Waking Up, LLC)—were selected based on their status as the top-grossing mental health apps in December 2022 [9], reflecting their commercial reach and user uptake rather than functional equivalence. While Calm, Headspace, and Waking Up all foreground guided meditation and mindfulness training as core components of user engagement, they differ notably in content and delivery approaches. Headspace adopts a structured, practical approach designed to make mindfulness accessible and habit-oriented, whereas Waking Up places greater emphasis on deeper philosophical discussions and the scientific underpinnings of consciousness [21,22]. Calm, by contrast, frames mindfulness within a broader relaxation and wellness context that incorporates sleep stories and ambient soundscapes alongside meditation guidance [23]. BetterSleep occupies a hybrid position, combining relaxation, soundscapes, and sleep-focused mindfulness with elements of habit formation, while Sleep Cycle is distinct in its emphasis on sleep tracking, biometric data, and algorithmic analysis, positioning mental well-being primarily through sleep optimization rather than direct psychological intervention [24,25]. These apps therefore differ substantially in their target users, behavioral change strategies, and technological affordances, ranging from content-driven experiential engagement to data-driven self-monitoring. A comparative analysis of these apps enables examination of how varied mHealth design logics, commercial priorities, and conceptualizations of mental health are operationalized within widely adopted platforms, providing insight into the broader mental health app market rather than a single therapeutic modality.

Social media offers a rich and ecologically valid data source for evaluating user experiences of apps. X (formerly Twitter), which allows third-party data access via its API, supports large-scale qualitative research by providing real-world, unsolicited feedback [26,27]. X’s arrival in 2006 predates that of any mental health apps by at least 2 years [28-30]. With around 238 million global monthly active users, the real-time and conversational nature of X results in over 80% of the platform’s advertisers’ inbound customer service requests arriving via the platform [31,32]. Unlike Facebook (developed by Meta Platforms, Inc) and Instagram (developed by Meta Platforms, Inc), which focus on connecting friends and sharing photos, X emphasizes text-based posts [33]. Given its functionality and comprehensive data archive, X was chosen as the data source for this study.

Natural language processing (NLP) has become an increasingly important set of methods in health research for extracting meaningful insights from unstructured text, supporting both clinical and patient-facing applications. NLP techniques have been applied across health care to analyze clinical notes, extract structured information from narrative records, and support decision-making processes, highlighting the role of text analytics in structuring and interpreting large volumes of health-related data [34]. In addition to clinical contexts, NLP has been used to analyze real-world patient and consumer language, including extracting health insights and patterns from free-text patient feedback and social media, demonstrating its utility in capturing perceptions, attitudes, and experiences related to health services and outcomes [35,36]. Recent work has shown how topic modeling, sentiment analysis, and related language processing techniques can complement traditional qualitative analyses by summarizing themes and sentiment patterns across large text datasets [35]. These applications provide context for this study’s use of NLP methods to analyze user-generated commentary on mental health apps.

Machine-assisted topic analysis (MATA) combines machine learning techniques with human-led qualitative analysis to enable systematic exploration of large-scale textual datasets. In this approach, free-text data are first structured using structural topic modeling (STM), making them analytically tractable while preserving the richness of user-generated content. MATA keeps human judgment central to topic labeling, thematic synthesis, and theoretical interpretation, rather than relying solely on automated classification. Building on established MATA applications in health research [37,38], this study extends the framework to the analysis of user experiences of commercial mental health apps expressed on social media. Methodologically, this implementation demonstrates how MATA can surface and organize diverse user concerns at scale—spanning technical usability, content and operational challenges, commercial practices, and self-monitoring experiences—that would be difficult to capture through traditional thematic analysis alone due to dataset size, or through sentiment analysis alone due to its limited contextual sensitivity.

In this study, STM was used to organize user feedback on mental health apps by identifying patterns of word co-occurrence while incorporating relevant metadata. Human-led thematic analysis was subsequently applied to interpret and cluster the resulting topics [37,39-44].

Focusing on user experiences rather than researcher-led evaluations, this study targeted the 5 high-grossing mental health apps in December 2022: Calm, Headspace, BetterSleep, Sleep Cycle, and Waking Up [9]. These apps were selected due to their widespread use, financial success, and potential to generate relevant, representative data. When searching for mental health apps, health or meditation app criteria were applied, while fitness, weight control, and sports apps were excluded. Table 1 briefly summarizes the 5 apps and their individual global revenue totals for December 2022.

**Table 1.** The top 5 grossing mental health apps in December 2022, their monthly revenue, and brief summaries.

App name	Approximate gross global revenue in the month of December 2022	Focus of app
Calm	6,400,000	Mindfulness, relaxation, and sleep
Headspace	4,900,000	Mindfulness, meditation, and sleep
Sleep Cycle	1,400,000	Sleep tracking and gentle wake-up alarm clock
BetterSleep	1,300,000	Sleep tracking, education, and relaxation content
Waking Up	952,000	Meditation, mindfulness, and educational content
Total	14,952,000	—

The above table shows the top 5 grossing mental health apps in December 2022, their monthly revenue, and brief summaries of each app [9,45-49].

The aim of this study was to identify issues users experience when using commercial mental health apps by analyzing a large volume of user-generated content from X. Specific objectives were: (1) to use MATA to analyze user feedback and (2) to provide insights on the potential negative impacts of highly popular commercial mental health apps.

## Methods

### Study Design

MATA combines topic modeling with human qualitative analysis [37,42-44]. In this study, MATA was used to analyze X posts, identifying topics and generating representative quotes. Qualitative researchers then interpreted the outputs to synthesize the findings.

### Data

X posts referencing the 5 highest-grossing health apps were collected using X’s official API, accessed via an academic license and implemented using version 0.3.1 of the R package “*academictwitteR*” [50] (refer to Table 1). The search strategy involved using keywords combining each app’s name with terms such as “app,” “application,” and “tracker,” where relevant. Additionally, X posts mentioning the official app accounts’ X handles were included in the search. A document showing the coding and keywords used is available in Multimedia Appendix 1. The date range for relevant posts gathered was set for X’s inception (March 21, 2006) to the end of 2022.

The number of posts related to each mental health app gathered using R from the initial dataset is shown in Table 2.

**Table 2.** Number of posts related to each individual app gathered using R from the initial dataset after negative sentiment filtering.

App	Number of X posts contributed to dataset
Calm	574
Headspace	9984
Sleep Cycle	5527
BetterSleep	912
Waking Up	2606
Total	19,603

### Preprocessing of the Data

Initially, 79,703 posts relating to the apps were gathered. The dataset then underwent negative sentiment lexicon filtering to identify posts expressing predominantly negative sentiment, consistent with the study’s focus on user-reported issues and concerns relating to mental health apps. Sentiment filtering was performed using the Bing lexicon implemented through version 0.3.1 of the *tidytext* package [50]. The Bing lexicon is a curated list of words annotated with positive or negative polarity. Each post was tokenized into individual words, which were then matched against the lexicon. Matched words were assigned a sentiment score of +1 (positive) or −1 (negative), whereas unrecognized words were excluded from scoring. These values were summed within each post to produce a net sentiment score, where negative values indicated an overall negative tone. Posts with a negative net sentiment score were retained for subsequent STM and qualitative thematic analysis. After negative sentiment filtering, 19,603 posts remained, and STM was applied to this dataset. Although this approach focused the analysis on expressions of negative user experience, some neutral or positive content remained within the final dataset, reflecting the inherent limitations of lexicon-based sentiment classification.

During additional data cleaning, app names were removed before STM was applied to organize the X posts into topics. Removing app names was crucial to prevent the STM from organizing X posts by name instead of the issues they presented. App names were reintroduced to the comments after STM had been completed. The data were preprocessed by first removing punctuation, symbols, and numbers, and then converting the text into individual token units. Common stop words were excluded, and stemming was applied to reduce words to their root forms, facilitating more efficient processing and analysis. In this study, no structured variables were included as covariates in the STM.

### Structural Topic Model

Before running the models, diagnostic plots were generated to determine the optimal number of topics, considering both the relevant metrics and the goals of this analysis, while focusing on balancing semantic coherence and exclusivity [37]. An unsupervised topic modeling approach was evaluated by human coders, testing models with differing numbers of topics (5-40) and differing covariates in terms of coherence, residuals, and interpretability. A structural topic model with 10 topics and 20 posts per topic was identified as the most suitable for gaining insights based on the diagnostic statistics. Ultimately, a model without covariates was chosen.

We tested multiple candidate models across a range of topic numbers and evaluated their quality using established STM diagnostics, consistent with the approach in Sheen et al [38]. Specifically, we examined semantic coherence (how strongly words in a topic co-occur) and topic exclusivity (how distinct words are across topics) to assess each model’s interpretability and distinctiveness. In addition to these quantitative diagnostics, candidate models were qualitatively reviewed by inspecting representative posts to determine whether the topics were meaningful and clearly interpretable. Based on this combined evaluation, a 10-topic model was selected because it provided the best balance: the topics were sufficiently distinct from one another (high exclusivity), internally coherent (high semantic coherence), and interpretable in terms of the substantive themes present in user comments on mental health apps. This approach mirrors the methodology used by Sheen et al [38], where the final number of topics was chosen based on both statistical diagnostics and qualitative interpretability rather than on a purely numeric criterion.

### Output

Each of the 10 topics included as part of the output analysis was represented by the 20 X posts identified as the most semantically related to a given topic. The intention of the STM approach used in this study was that the posts within each topic would share commonalities because of the word co-occurrence of X posts within the generated topics, with the human researchers subsequently being able to label each topic by describing the main user issue evidenced by each topic. All 10 topics and quotes are provided in Multimedia Appendix 2.

### Qualitative Analysis Process

After establishing the structural topic model, manual thematic analysis was performed by two researchers—Jack Bolter (JB) and an additional researcher—to provide interpretation of the topics. The researchers independently described each quote and labeled the STM-created topics based on the main issue raised by each topic. After independent analysis, the researchers discussed and agreed upon topic labels. A third researcher (PB) reviewed the analysis to increase the quality of the analysis. Where appropriate, the researchers organized the topics into major themes. Representative quotes for each topic are discussed in the Results section.

The following sequential steps describe how the researchers conducted the analysis following receipt of the STM output:

- Step 1: the researchers familiarized themselves with the data through repeated readings of the machine-generated topics and noted descriptions of each X-post.
- Step 2: the researchers independently added labels to describe each of the machine-generated topics.
- Step 3: the researchers discussed and reached consensus on major themes in their analyses, considering similarities and differences.
- Step 4: the researchers’ findings and interpretations were documented in the Results and Discussion sections.

This study used MATA, a mixed methods approach that combines computational text analysis with qualitative interpretation to identify patterns in large text datasets. Following Bondaronek et al [37], MATA involves the application of automated topic modeling techniques to structure a large corpus of textual data into algorithmically derived topic groupings. These outputs are then reviewed and interpreted by researchers, who iteratively generate topic labels and develop higher-order themes. Rather than replacing human interpretation, the computational component is used to facilitate systematic exploration of large datasets, while interpretive analysis remains central to theme development and meaning-making.

While no automated bot or spam detection filters were applied during data collection, several aspects of the analytic process acted as a form of natural mitigation against the influence of such noise. Posts relating to the selected mental health apps were first organized using STM, which groups content based on patterns of semantic similarity. Given that bot or spam posts are often repetitive, promotional, or thematically inconsistent, such content is less likely to cohere meaningfully into stable topics.

No software was used to conduct the subsequent manual thematic analysis. Two researchers (JB and an additional researcher) independently reviewed the STM-generated topics and developed initial topic labels through close reading and interpretation of the data. An inductive approach was used for thematic labeling, with topic labels and themes generated directly from researcher interpretation of the STM outputs. The labels generated for each topic were based on the overall consistency and substantive meaning of the posts within a topic. These independently generated labels were then compared and discussed. Any minor differences in interpretation were resolved through discussion and consensus. The researchers’ interpretations were closely aligned, and discussion primarily served to refine and harmonize topic labels and overarching themes based on shared review of the STM outputs. Topic interpretation was therefore driven by recurring, coherent user experiences rather than individual posts. One topic was excluded entirely due to low coherence, illustrating how the human interpretive stage helped identify and remove clusters lacking a clear thematic thread, which may include random or spam-like content. In addition, not all posts within a topic contributed equally to theme definition; more detailed and thematically consistent posts were prioritized during interpretation, reducing the influence of isolated or anomalous content. While it remains possible that some bot or spam posts were present, industry estimates suggest such content accounted for approximately 10% of posts on X during the study period [51], indicating that any residual noise likely constituted a small minority and did not drive the overarching thematic findings.

### Ethical Considerations

The permission to conduct the study was granted by The University of Westminster’s Ethics Committee under application ID ETH2223-2307 (class 1). Users consent to X redistributing their public comments on its platform upon signing up to the platform [28]. To protect privacy, identifying details such as names and X handles were removed from reported results.

Beyond ethics approval and platform terms of service compliance, measures were taken to minimize potential harm associated with sensitive mental health-related content, including anonymization of usernames and handles. Results were primarily reported at the level of topics and themes, with a limited number of anonymized posts included as illustrative examples to support interpretation.

## Results

### Overview

Following data cleaning and negative sentiment filtering, 19,603 app-related X posts remained for STM topic organization (79,703 posts were gathered relating to the apps prior to negative sentiment filtering). Eight of the 10 subsequently STM-generated topics were included in the analysis, as these topics were coherent and provided insights relating to the issues experienced by users of the apps. The remaining 2 topics organized by the model were excluded from the analysis, 1 due to incoherence and the other due to comments being related to social media content rather than the apps themselves. As per Bondaronek et al [37], the researchers coded the X posts and assigned a label to each topic which summarized the main user issue identified. Eight of these topics were then organized into 4 themes (1 theme comprised a single stand-alone topic).

**Figure 1.**
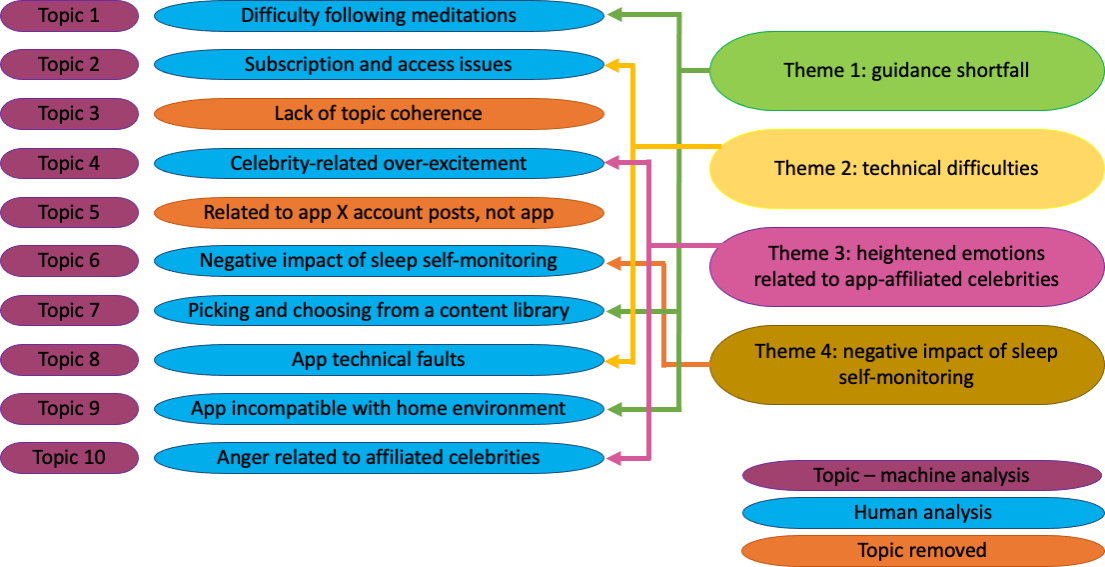
Overview of the themes and topic labels derived from the structural topic model and interpreted through human thematic analysis by JB and an additional researcher. The guidance shortfall theme captures users’ difficulties engaging with audio-guided content, including challenges understanding meditation instructions, selective and repetitive content use, and incompatibility with busy home environments. Technical difficulties reflect frustrations related to subscription access, in-app functionality, and limited responsiveness from app support services. Heightened emotions related to app-affiliated celebrities represent both positive excitement and negative reactions to celebrity involvement in app content and promotion. The negative impact of sleep self-monitoring theme highlights users’ reliance on app-based sleep tracking, including benefits for some users alongside indications of reduced sleep self-efficacy and increased anxiety linked to monitoring practices.

Figure 1 shows each of the topic labels and themes that the researchers produced to organize and derive meaning from the STM-generated topics.

### Guidance Shortfall

The “guidance shortfall” main theme clustered 3 topics in which user comments demonstrated that additional guidance beyond the content within the apps would have helped users with their mindfulness and sleep hygiene practices.

Topic 1 focuses on users’ difficulty following the apps’ audio-guided meditations. Some quotes indicated trouble interpreting metaphors and concepts in the guided exercises, which appeared to hinder practice comprehension, while others demonstrated that users were physically uncomfortable while following the exercises.

*I’m struggling with the idea of awareness in space and then being aware from it. I try to imagine it but all my awareness comes from my brain. I can imagine my awareness intermingling with space but in reality my awareness is only acknowledging space as aware from my brain.*

[Topic 1, 18]

Topic 7 illustrates how users take the opportunity to pick and choose content from these apps and may use the same pieces of content repeatedly as opposed to following a structured course. Comments often appeared to be in response to posts from the apps’ social media accounts asking X users what their favorite pieces of content on their respective apps were. For example, the following comment highlights one user’s preference for two sleep podcasts on one of the apps:

Moon Buggy or Rainy Day Antiques... for the ZZZZs....

[Topic 7, 10]

Topic 9 was characterized by users commenting that they found their busy home environments to be incompatible with the apps. For example, one user reported difficulty following the relaxation and meditation guidance of the apps while they were aware of other noises within their home.

*Tonight’s episode was accompanied by the sounds of the hamster trying to break out of his cage, 6yr old’s toy tiger roaring & 3yr old having coughing fit.*

[Topic 9, 20]

Topic 9 also illustrated how busy home environments can interfere with recordings used to provide sleep quality analysis.

*My [app name] app is supposed to record me snoring but most of the recordings are of (R)ingo snoring or him hissing at (B)owie.*

[Topic 9, 5]

### Technical Difficulties

The “technical difficulties” theme comprised 2 topics describing how users experienced technical problems when trying to access the apps and while using the apps.

Topic 2 was focused on subscription and access issues. Many users showed frustration due to not having gained the access levels they are entitled to despite having paid their subscription or being due access for another reason (eg, promotions allowing app access to individuals in certain professions).

*… I’m confused. I paid £49.99 annual subscription but I still have some areas locked. Why is this please(?).*

[Topic 2, 2]

Such frustration was exacerbated when users struggled to contact app staff to seek support or compensation.

*(I have) been trying to contact you for several days by email. No response from your team. Very disappointed. My subscription fee charged was a lot higher than what was shown in−app. Please reverse this immediately...*

[Topic 2, 16]

Topic 8 highlighted that some users experienced negative emotions due to technical faults encountered while using the apps.

*I’m confounded by how LOUD the default volume is on music, SFX, stories, etc ... with no apparent way to adjust the volume, in app. If the answer=turn down iPhone volume, then alarm volume is compromised. Unusable. Am I missing something?*

[Topic 8, 18]

As with topic 2, such frustration was exacerbated when users struggled to contact app support staff:

*How do I escalate a fault on my app which I raised on 5 Aug(?) (It is) with the engineers but “Sadly, we don’t currently have an estimate for when the issue might be fully resolved(.)”*

[Topic 8, 20]

### Heightened Emotions Related to App-Affiliated Celebrities

The main theme related to “app-affiliated celebrities” clustered 2 topics showing how users experienced heightened emotions—both positive and negative—about public figures with links to the apps. Such affiliations were generally due to celebrities either creating content for the apps or having sponsorship deals for the apps on their own platforms.

Topic 4 demonstrated that celebrity partnerships with the apps can have an effect of elevating users to states of excitement that are counter to the states of calm and relaxation on which the apps are marketed. Several X posts were all in capital letters and related to an appearance of a famous popstar on one of the apps to narrate content.

*OMFG, I’m dead. The shoes not even on his feet. His hat the beard his outfit. OMG.*

[Topic 4, 19]

While comments in topic 4 were largely positive, if perhaps excitable in tone, those in topic 10 often expressed anger in relation to celebrities affiliated with the apps.

*(Celebrity name) (is) a fraud owned by China… the most overrated player in NBA history.*

[Topic 10, 3]

### Negative Impact of Sleep Self-Monitoring

Topic 6 is a stand-alone theme relating to the reliance some users seemed to have on using these apps to aid their sleep and the sleep self-monitoring they performed via the app. Many of the user comments were positive about how the apps impacted their sleep.

*The (app name) App for iOS is a game changer. Wakes you up at the end of a cycle so as to not startle you awake from REM sleep.*

[Topic 6, 3]

However, other posts hinted that users’ focus on app statistics might negatively impact their sleep self-efficacy and be problematic due to excessive sleep self-monitoring.

*Ok party people, I’m totally addicted and fascinated by the (app name) app! Always knew I sucked at sleeping but WOW my graphs r shocking!*

[Topic 6, 14]

## Discussion

### Principal Findings

This study investigated issues users experience with mental health apps by analyzing 19,603 negative sentiment X posts related to the 5 highest-grossing mental health apps (79,703 posts relating to the apps were gathered prior to negative sentiment filtering). Using machine learning with qualitative analysis, the data were organized into 10 topics and interpreted by researchers into four main themes: guidance shortfalls, technical difficulties, heightened emotions linked to app-affiliated celebrities, and the negative impact of sleep self-monitoring. These themes encapsulated a range of richly detailed user concerns, including frustration with insufficient in-app guidance, technical obstacles, overexcitement and distress related to celebrity presence, and a potentially detrimental overemphasis on sleep data. Although users did not report direct feelings of unsafety, the absence of in-person support and the intensity of negative emotions expressed suggest that some users perceived limitations in the ability of unregulated mental health apps to meet their support needs.

Several findings from the study align with existing research. Users’ difficulties with guided meditation and feelings of being unsupported resonate with Crane et al [52], who emphasize the importance of instructor feedback in mindfulness practice via the interactive inquiry section of classes, and Ruse et al [53], who report dissatisfaction stemming from the lack of human interaction in mental health apps. Users’ perceptions of feeling unsupported by audio guidance during guided meditations can be interpreted through self-determination theory, which posits that sustained motivation depends on supporting basic psychological needs such as autonomy and competence; when digital guidance fails to scaffold these needs, users are less likely to internalize the practice and maintain engagement. Self-determination theory has been applied in human-computer interaction research to explain how technology design can either foster or undermine intrinsic motivation by shaping whether users feel capable, self-directed, and meaningfully engaged [54]. While audio guidance is intended to support practice, limited personalization and adaptive feedback—which are more feasible in in-person meditation classes and groups—may diminish users’ sense of autonomy and competence, reducing the subjective feeling of being supported. Similarly, the observation that technical issues led to frustration and stress supports findings by Haggag et al [55] and Woods et al [56], who highlight usability problems as key barriers to engagement and therapeutic benefit. The study’s insights into the risks of sleep self-monitoring—such as anxiety over sleep statistics and potential overfocus on tracking—also reflect earlier research by Baron et al [57] on orthosomnia (the idea that preoccupation with sleep quality and tracking results may paradoxically impair sleep quality).

Recent research has critically examined the user experience and interpretive practices associated with sleep tracking technologies, highlighting issues of data literacy, accuracy of app data, and optimization logic assumptions. O’Neill and Nansen [58] analyzed how mobile sleep apps do not simply monitor sleep but actively intervene in users’ sleep-wake rhythms through features such as “smart wake-up” alarms and brainwave entrainment sounds. They show how these functions are underpinned by optimization logics that position sleep as something that can be measured, managed, and improved through algorithmic systems, thereby shaping how users understand and act upon their own sleep. The authors illustrate that this is problematic given the potential for these apps to produce inaccurate user sleep data. Similarly, Liang et al [59] demonstrate that users often grapple with interpreting sleep data, questioning its reliability and struggling to translate metrics into meaningful or actionable insights. Together, this literature underscores that engagement with sleep tracking apps involves complex processes of interpretation, negotiation, and trust. However, it also demonstrates that such trust can also generate user anxiety that they are not achieving optimal sleep.

In terms of novel contributions, this study found that users expressed strong emotional responses to celebrity content within mental health apps, which some posts suggested could detract from experiences of calm, focus, and emotional regulation that users associated with mindfulness practices. Existing research related to celebrity affiliations with mHealth apps has largely focused on the commercial benefits rather than their potential to interfere with therapeutic outcomes [60]. This study also highlights how apps’ associations—or disassociations—with public figures can provoke strong emotional responses in users, including anger, particularly when these public figures are involved in polarizing discourse. This suggests that app branding and celebrity affiliations may be associated with socially and politically charged reactions among users. While prior work has explored the marketing benefits of celebrity endorsement, the psychological and affective implications for users remain underexplored in the mHealth app literature. The findings relating to celebrity affiliations for commercial purposes contribute to a growing body of literature that critiques the ways in which digital wellness and mindfulness apps draw from traditional Eastern philosophies—such as Buddhist practices rooted in ethical precepts such as sila—while stripping away their moral and communal dimensions [61-63]. These practices follow other recent examples of traditional mindfulness practices being reframed within Western commercial frameworks to promote individual calm, emotional regulation, or productivity, raising questions about cultural appropriation and the commodification of spirituality.

It was also found that some users repeatedly engage with familiar or favorite content. Such repeated use of the same content as opposed to following a structured program may mean users are less likely to experience meaningful behavior change. While Bol et al [64] found that autonomy and customization when using mHealth apps can increase behavioral intentions for users with a high need for autonomy, their study also reported that, on average, customization did not lead to greater perceived control, motivation, or intention to act. While user autonomy may facilitate short-term engagement, long-term behavioral change may require more structured, theory-driven approaches, such as those informed by the Health Action Process Approach [65]. Further research is warranted to clarify the conditions under which user autonomy supports or hinders behavioral outcomes in mHealth app contexts.

### Strengths and Weaknesses

A significant strength of the study lies in its methodological approach, using STM in combination with qualitative analysis to effectively synthesize insights from a vast volume of real-world data. This approach, as demonstrated by Bondaronek et al [37], proved reliable and valid. The use of X posts enhanced ecological validity by capturing unfiltered user experiences in situ [26].

A key limitation of this study lies in the lack of nuance inherent in the methodological approach used. While STM is effective for identifying broad thematic patterns across large datasets, it is not well suited to capturing the complexity and depth of individual user experiences. This is compounded by the low character limits of most X posts within the available data, which constrain user expression and may exclude more detailed or reflective insights. Moreover, the diversity of the apps analyzed—ranging from mindfulness-focused to sleep-oriented platforms—introduces variability that may limit the generalizability of specific findings across the broader category of mHealth apps. Future research could address these limitations through follow-up interviews or focus groups to help verify the findings and gain a richer understanding of the identified user experiences.

The absence of automated spam or bot detection represents a limitation of this study, as no specific bot-detection procedures were applied to the dataset prior to analysis. Consequently, the STM input data included a degree of automated or low-quality content, introducing noise into topic summarization and contributing to some less coherent or contextually relevant posts within topics. Although spam and bot detection methods may have reduced this noise and potentially improved topic coherence and interpretability, such approaches are imperfect, particularly when applied to large-scale social media datasets, meaning that some automated content may still have remained even if filtering had been undertaken. To minimize the influence of noisy data on interpretation, the analysis focused on topics that demonstrated coherence and relevance to user experiences, with an incoherent topic and another unrelated to user experiences being excluded from the subsequent analysis.

Another key limitation of this study is that it draws on publicly shared social media posts, and we cannot infer that users’ experiences reported online correspond to clinically assessed mental health outcomes. The themes identified reflect users’ self-reported issues when using mental health apps, rather than verified experiences of anxiety or other clinical conditions. Consequently, the findings should be interpreted as indicative of the types of issues users report, rather than evidence of therapeutic effectiveness or harm.

It is also important to recognize that X users are not fully representative of the broader population of mental health app users, which may limit the generalizability of our findings. Demographically, X’s user base skews toward younger adults, with an estimated 71.6% of users being <34 years in 2025. The X user base is also male-dominated, with males comprising 63.7% of the global users in 2025 [66]. Furthermore, for the time period from which the data were gathered (2006 up until the end of 2022), X users tended to be more highly educated and comparatively affluent than the general population, with higher proportions of adults in higher income brackets and tertiary education levels [67]. These demographic profiles suggest that perspectives captured in our analysis may overrepresent the experiences of younger, more educated, and higher-income individuals, while underrepresenting older adults, lower-income groups, and those who do not engage with X as a forum for discussing app experiences.

Character limits were only increased to 4000 characters for paying subscribers after this study’s data were collected, meaning that X posts in this study had character limits of 140 (for X posts posted prior to November 6, 2017) and 280 (for X posts posted from November 7, 2017, onward) [68]. Such brevity was advantageous for the manageability of the dataset, but it also limited the extent to which users were able to articulate their experiences.

The dataset was unevenly distributed across the 5 included mental health apps, with some apps generating substantially more posts than others. This variation reflects differences in app popularity and user engagement on the platform, rather than a sampling bias introduced by the study design. However, this imbalance may have influenced the relative prominence of topics identified through STM, as apps with higher volumes of user-generated content are potentially more likely to contribute greater proportions of text to the corpus. The study was designed to identify common issues associated with mental health app use more broadly, rather than to conduct app-specific comparisons, and therefore findings should be interpreted at this aggregate level. No sensitivity analyses were conducted to rebalance the dataset or isolate app-specific effects, as this was beyond the scope of the study’s exploratory aims.

### Implications for Research, Practice, and Policy

App developers should address the lack of guidance support identified in user reports by strengthening onboarding processes, in-app guidance, and wayfinding features. Difficulties navigating app content and uncertainty about how to engage with interventions suggest a need for clearer instructional scaffolding and more explicit signposting of core features. To ensure such guidance supports meaningful behavior change rather than surface-level engagement, interventions should be aligned with established behavior change theories such as the Health Action Process Approach [65].

User complaints relating to subscription practices and billing indicate a need for greater transparency around pricing structures, trial periods, and cancellation processes, as well as more responsive customer support mechanisms. Similarly, reports of technical instability and disrupted functionality point to the importance of improving app reliability to prevent frustration and disengagement. Together, these issues suggest that usability and commercial design decisions may contribute to users reporting frustration, stress, and feelings of being unsupported, potentially diminishing the perceived value of mental health apps.

The findings of this study suggest that greater consideration should be given to the regulation and oversight of mental health apps. Policymakers should consider frameworks to evaluate and certify digital mental health tools, especially those that might be used in place of traditional therapeutic support. Certification schemes could distinguish evidence-based digital therapeutics from general wellness apps. Dialogue between public health bodies and app providers, as well as public information campaigns, may help users make more informed choices about mental health support and whether they should use mental health apps. Clinicians could also use the present findings to support patients in critically evaluating app-based mental health tools and, where appropriate, integrate curated lists of validated apps into care strategies to complement—not replace—traditional therapeutic support.

Future research should test the robustness of this study’s STM-based findings across diverse populations and mental health contexts. Additional studies could explore whether key user issues were missed due to the analytical approach of STM. Research is needed to evaluate the psychological outcomes associated with mental health app use and to explore users’ perceptions regarding the role of these apps alongside, or potentially in place of, professional support.

### Conclusion

This study provides further evidence that STM can be combined with human qualitative methods to quickly analyze large volumes of X data. With data relating to the 5 most popular mental health apps analyzed via this approach, the researchers were able to expeditiously identify several issues encountered by users. This method, therefore, has the potential to be used to swiftly provide insights relating to how individuals experience various digital health interventions.

## Supplementary material

10.2196/85575 Multimedia Appendix 1 Document illustrating the coding and keywords used to gather X posts relating to the 5 top-grossing mental health apps.

10.2196/85575 Multimedia Appendix 2 All topics with redactions.

## Abbreviations

MATA: machine-assisted thematic analysis
mHealth: mobile health
NLP: natural language processing
STM: structural topic modeling
