# Supplementary material for: User-Reported Issues With Mental Health Apps: Machine-Assisted Topic Analysis of Social Media Posts
Source: JMIR Mhealth Uhealth. 2026 Jul 21;14:e85575. doi: 10.2196/85575 (PMC13388529; doi:10.2196/85575)
Supplement: Multimedia Appendix 1 [file mhealth-v14-e85575-s001.docx]

**Document illustrating the coding and keywords used to gather tweets relating to the 5 top-grossing mental health apps**

## install + load packages

#install.packages("academictwitteR")

#install.packages("tidyverse")

library(academictwitteR)

library(tidyverse)

setwd("~/Documents/Apps safety")

## set bearer token

set_bearer()

## query API for tweets

## helpful query docs here:

## https://developer.twitter.com/en/docs/twitter-api/tweets/search/integrate/build-a-query#availability

# Mental top 5

calm <-

get_all_tweets(

query = "calm app OR calm application OR calm meditation",

start_tweets = "2012-05-01T00:00:00Z",

end_tweets = "2023-01-01T00:00:00Z",

lang = "en",

data_path = "data/calm/",

n = 100000,

bind_tweets = F

)

headspace <-

get_all_tweets(

query = "headspace app OR headspace application OR head space app OR head space application OR headspace",

start_tweets = "2010-05-02T00:00:00Z",

end_tweets = "2023-01-01T00:00:00Z",

lang = "en",

data_path = "data/headspace/",

n = 100000,

bind_tweets = F

)

sleepcycle <-

get_all_tweets(

query = "sleepcycle app OR sleepcycle application OR sleep cycle app OR sleep cycle application OR sleep cycle clock

OR sleepcycle clock OR sleep cycle alarm OR sleepcycle alarm",

start_tweets = "2009-01-01T00:00:00Z",

end_tweets = "2023-01-01T00:00:00Z",

lang = "en",

data_path = "data/sleepcycle/",

n = 100000,

bind_tweets = F

)

ipnos <-

get_all_tweets(

query = "ipnos app OR ipnos application OR bettersleep app OR bettersleep application OR

better sleep app OR better sleep application OR bettersleep tracker OR bettersleep tracker OR

better sleep tracker OR better sleep tracker OR ipnos tracker",

start_tweets = "2009-05-07T00:00:00Z",

end_tweets = "2023-01-01T00:00:00Z",

lang = "en",

data_path = "data/ipnos/",

n = 100000,

bind_tweets = F

)

wakingup <-

get_all_tweets(

query = "wakingup app OR wakingup application OR wakingup meditation OR

waking up app OR waking up application OR waking up meditation OR sam harris app OR sam harris application",

start_tweets = "2018-09-01T00:00:00Z",

end_tweets = "2023-01-01T00:00:00Z",

lang = "en",

data_path = "data/wakingup/",

n = 100000,

bind_tweets = F

)
