# Supplementary material for: User-Reported Issues With Mental Health Apps: Machine-Assisted Topic Analysis of Social Media Posts
Source: JMIR Mhealth Uhealth. 2026 Jul 21;14:e85575. doi: 10.2196/85575 (PMC13388529; doi:10.2196/85575)
Supplement: Multimedia Appendix 2 [file mhealth-v14-e85575-s002.docx]

***Topic 1***

| *OZENS OF POLICE CHIEFS, SHERIFFS CALL ON TRUMP TO END GOVERNMENT SHUTDOWN AND FORGET BORDER WALL! Law enforcement officials warned that the shutdown h circumstances that "threaten public safety." The Letter was signed by 44 police chiefs and sheriffs across the country*  *-------------------------------------------------------------------------------------------------------------------------------------*  *█████ Absurd as it is but it...s a real drag to try to pull off Tomas Hardy...s having no head technique wile wearing glasses , embrace the blur I suppose.*  *-------------------------------------------------------------------------------------------------------------------------------------*  *█████ "To thine own self be true. Thou canst not then be false to any other man." [Polonius advice to Laertes; Shakespeare's Hamlet.]*  *-------------------------------------------------------------------------------------------------------------------------------------*  *█████ To ressource myself nothing beats to "my calm" loneliness and isolation when stress overwhelms me constraints begin to overwhelm me and I feel my energy supply diminish the urge to cut ties with the outside world is quickly felt...(breath relax and meditate)......*  *-------------------------------------------------------------------------------------------------------------------------------------*  *█████ I have trouble when you instruct me to superimpose an image onto my visual field. Of course I have a visual field, and of course I can imagine visual objects, but I don't seem to be able to superimpose those imagined objects onto the same visual field ...*  *-------------------------------------------------------------------------------------------------------------------------------------*  *█████ The neural pathways that manage stress overlap greatly w/ neurons that manage physical pain (see vagal nerve), so managing stress directly impacts our experience of pain. The █████ app has a nice series on managing pain, and binaural beats work for many 2*  *-------------------------------------------------------------------------------------------------------------------------------------*  *█████ how should distracting anxiety−related physical symptoms eg globus, nausea be dealt with during meditation? Thanks*  *-------------------------------------------------------------------------------------------------------------------------------------*  *█████ I often wonder which of my beliefs are totally wrong. Searching for and discovering these is one of the primary goals of learning and thinking. This is painful but necessary.*  *-------------------------------------------------------------------------------------------------------------------------------------*  *█████ Walking, moving, breathing, stretching, mindfulness, challenging cognitions about pain downregulate it! Tips from pain management ................ try the █████ app pain section*  *-------------------------------------------------------------------------------------------------------------------------------------*  *█████ Being in nature lowers your stress level hormone, reduces your heart rate and blood pressure. Go get some air outside!*  *-------------------------------------------------------------------------------------------------------------------------------------*  *█████ Why do guided meditations always want me to hold me breath?! That is so uncomfortable and so unnatural to me*  *-------------------------------------------------------------------------------------------------------------------------------------*  *█████ re: having no head − It makes a lot of sense to me but I am distracted by seeing the frames of my glasses, my brain wants to perceive them as evidence of having a head. should I view the glasses as a mysterious inanimate object shape? should I take them off?*  *-------------------------------------------------------------------------------------------------------------------------------------*  *Buzzwords. Cognitive dissonance. Internalising. Cultural appropriation. Self awareness. Grounded. Sorry I'm meditating with my █████ app*  *-------------------------------------------------------------------------------------------------------------------------------------*  *█████ I disagree, it...s easy to be kind to others but waaay more difficult to be kind to yourself*  *-------------------------------------------------------------------------------------------------------------------------------------*  *█████ I'm sorry you're going through it! Yung best advice na nakuha ko tlga was "keep the anger moving". Kasi if it stagnates it burns you &amp; ppl who don't deserve it. Iwas Targaryen madness. Meditation helps a lot (tinodo ko lang yung █████ app) + occasional therapy as needed.*  *-------------------------------------------------------------------------------------------------------------------------------------*  *█████ It's obvious the federation is more worried about their tradition than the players. I think the players all should walk out &amp; not play any of the Grand Slam tournaments, that would get their panties all in a bunch with those pompous snobs who run the tournaments.*  *-------------------------------------------------------------------------------------------------------------------------------------*  *█████ So sorry. I...ve endured horrific pain before. Couple ideas: █████ App − there...s a pain meditation series w/in it. (A soldier with body parts blown up/chronic pain says meditation better than meds.) Turmeric for anti−inflammatory. Fresh pineapple for pain. Comedy for mindset.......*  *-------------------------------------------------------------------------------------------------------------------------------------*  *█████ I...m struggling with the idea of awareness in space and then being aware from it. I try to imagine it but all my awareness comes from my brain. I can imagine my awareness intermingling with space but in reality my awareness is only acknowledging space as aware from my brain.*  *-------------------------------------------------------------------------------------------------------------------------------------*  *█████ During today's meditation I felt an odd tension arise. While I was observing my visual field I attempted to look for myself. Almost immediately a bodily tension arose. My muscles were relaxed but my insides were tense. It felt pleasant but otherwise inexplicable.*  *-------------------------------------------------------------------------------------------------------------------------------------*  *█████ When the headaches subside, moments of being pain−free when Nature coaxes me into breathing its air and covers me in its shade.* |
| --- |

***Topic 2***

| *█████ Are you guys having issues with the gift sending? Quite frustrating that the email didn't arrive on the date requested, and the link to check order status is broken, and the order confirmation email doesn't list the date.*  *--------------------------------------------------------------------------------------------------------------------------------------------*  *█████ █████ hi I'm confused. I paid £49.99 annual subscription but I still have some areas locked. Why is this please.*  *--------------------------------------------------------------------------------------------------------------------------------------------*  *█████ █████ I bought the spotify combo. Unable to login. Error: Already linked my email to the free Headspace. Please advise.*  *--------------------------------------------------------------------------------------------------------------------------------------------*  *█████ hi, I received the email with 30 days free but link says ...internal server error... emailed you guys and you replied quickly, but only to send me the same link with the same ...internal server error... page. Please help, thanks*  *--------------------------------------------------------------------------------------------------------------------------------------------*  *█████ hi, I'm having trouble on all devices buying subscription. 'confirm payment' button seems inactive. Is this known problem? Thanks*  *--------------------------------------------------------------------------------------------------------------------------------------------*  *█████ hi there, I've been trying to buy a gift subscription from the █████ website but it's buggy and won't let me to login to my existing Facebook−based █████ account. Because the FB login button on the website is totally unresponsive.*  *--------------------------------------------------------------------------------------------------------------------------------------------*  *█████ Hi █████, having problems with my account. Had signed up for free premium membership offered to doctors, but my account keeps asking me to try ppremium membershi Very frustrating as cannot access most features. Help please*  *--------------------------------------------------------------------------------------------------------------------------------------------*  *█████ 503 error when I try to cancel my sub, and missing email field to contact support. Sneaky. But srsly, contact me to cancel pls..*  *--------------------------------------------------------------------------------------------------------------------------------------------*  *█████ my wife has been charged £45.78 for a subscription she was not aware of. It...s impossible to detect how to cancel a subscription on your website. It...s deliberately confusing!*  *--------------------------------------------------------------------------------------------------------------------------------------------*  *█████ hey guys I sent you an urgent support email − I was given an inappropriate human−readable gift subscription code! Need it for xmas!*  *--------------------------------------------------------------------------------------------------------------------------------------------*  *█████ █████ logged me out due to an 'unexpected error' when I try to log back in through facebook it says 'authentication failed'?*  *--------------------------------------------------------------------------------------------------------------------------------------------*  *█████. Unhappy that my subscription has renewed and you won...t reply to my numerous emails asking for my money back. Disgusting service from █████ !!!!*  *--------------------------------------------------------------------------------------------------------------------------------------------*  *█████ having a huge problem cancelling your service and i'm not finding any contact number on your website to speak with someone. What is your customer service number?*  *--------------------------------------------------------------------------------------------------------------------------------------------*  *█████ are there any current discount codes for an annual subscription I did have an email from a few months back but it...s expired now and want to sign up thanks*  *--------------------------------------------------------------------------------------------------------------------------------------------*  *█████ I received a special offer email yesterday with an already expired code. Any chance of an up to date code so I can sign up?*  *--------------------------------------------------------------------------------------------------------------------------------------------*  *█████ been trying to contact you for several days by email. No response from your team. Very disappointed. My subscription fee charged was a lot higher than what was shown in− app. Please reverse this immediately. █████*  *--------------------------------------------------------------------------------------------------------------------------------------------*  *█████ your mobile login flow should treat Facebook login the same if it logs in or creates a new account. Currently errors if you attempt to signup with a FB account that already associated with an account. Should just login*  *--------------------------------------------------------------------------------------------------------------------------------------------*  *█████ my subscription expired and yet I got no email or notification. What a lack of customer service.*  *--------------------------------------------------------------------------------------------------------------------------------------------*  *█████ My subscription expired, and when I try to renew, I get ...Cannot connect to the iTunes Store... error. Was charged, can...t activate.*  *--------------------------------------------------------------------------------------------------------------------------------------------*  *█████ please respond to my emails. I have had to open a dispute with paypal for the misleading billing info and nobody has got back to me!* |
| --- |

***Topic 3***

| *█████ Bi−polar is used by DSM as a legitimate disorder. There are no terms to describe it*  *------------------------------------------------------------------------------------------------------------------------------------------------------------------------*  *█████ I had breast cancer and I...m currently awaiting surgery for a large mass on my ovary (we won...t know what it is until it...s out). I...m finding the █████ app really useful at the moment, it has a course for cancer sufferers. Wishing you luck with your treatment ......*  *------------------------------------------------------------------------------------------------------------------------------------------------------------------------*  *█████ The █████ app is free to all educators − one of the assistant principals at my school got me into it. I was super sketchy about it initially because I despise the idea that ...organic yoga crystals lulu lemon essential oils...fix people*  *------------------------------------------------------------------------------------------------------------------------------------------------------------------------*  *█████ Also a soundscape of the harbour during a storm − crashing waves, wind, clanking of sailing boats ropes on the masts, seagulls etc.*  *------------------------------------------------------------------------------------------------------------------------------------------------------------------------*  *█████ Because it's illegal to beat the crap out of the people ruining my sunny zen−like disposition*  *------------------------------------------------------------------------------------------------------------------------------------------------------------------------*  *█████ An outdoor skating pond that we stumbled upon today ...*  *------------------------------------------------------------------------------------------------------------------------------------------------------------------------*  *█████ my pinned thread of threads has a full history of my practice, but most immediately preceding the sensitivity issues was Acem (non−directive, TM−like), but I also started using █████ app (Vipassana with a splash of dzogchen I guess?) and practicing Mett.. while unraveling*  *------------------------------------------------------------------------------------------------------------------------------------------------------------------------*  *█████ why stop the usage of █████ with Alexa. Myself and daughter use a lot. And now my daughter can...t use █████ o her echo dot as I won...t let her have a device in he room due to the temptation. Please change this. I can...t justify renewing █████.*  *------------------------------------------------------------------------------------------------------------------------------------------------------------------------*  *Ughhhh power nap then lab at 1 fuck meee. Def using my █████ alarm. #tired*  *------------------------------------------------------------------------------------------------------------------------------------------------------------------------*  *.... 75% of women have experienced low sex drive. .... 9 out of 10 women think that the medical industry doesn...t take women...s desire seriously. ............... Only 27% of women talk to their doctor about their sex life ...... 63% of women experience anxiety when it comes to their sex life.*  *------------------------------------------------------------------------------------------------------------------------------------------------------------------------*  *█████ Yep, today I chose to be lazy ........ for various reasons*  *------------------------------------------------------------------------------------------------------------------------------------------------------------------------*  *█████ Kati Morton has been proven by multiple Dr...s to be using outdated terms and information and to be falsely misleading people about ASPS. She is also proven to have lied about advice given to help someone with an eating disorder and enabling said eating disorder later on*  *------------------------------------------------------------------------------------------------------------------------------------------------------------------------*  *█████ Unfortunately I had to drop out of the group meditation today because the introduction was so badly phrased that it upset me. As a queer person, to be told by a privileged white man that labels aren't useful and labels do not matter, makes me feel silenced and ignored.*  *------------------------------------------------------------------------------------------------------------------------------------------------------------------------*  *The stresses of modern−day life tends to cause it to operate in a heightened state of alert, being triggered by small stressors and major stressors.*  *------------------------------------------------------------------------------------------------------------------------------------------------------------------------*  *I bet if everyone used a █████ app, a lot less people would hate their jobs*  *------------------------------------------------------------------------------------------------------------------------------------------------------------------------*  *█████ with Richard Gere today and Kung Fu Panda the other day, is █████ on some kind of weird Netflix binge?*  *------------------------------------------------------------------------------------------------------------------------------------------------------------------------*  *█████ Our clinging to ever−changing life is the main reason of suffering.*  *------------------------------------------------------------------------------------------------------------------------------------------------------------------------*  *█████ I...m dumping you. You dumped Ben Shapiro which is hostile to calm. Adios! Everyone that follows me−please do the same!*  *------------------------------------------------------------------------------------------------------------------------------------------------------------------------*  *█████ talking about digital mindfulness based intervention for people with #asthma #SWSAPC19 Used █████ app. Feasibility trial, initially in Lifeguide but problems associated with 2 digital platforms. Able to monitor app use. 158 people, from 17 practices.*  *------------------------------------------------------------------------------------------------------------------------------------------------------------------------*  *█████ How ABSURD is it for @calm to use such a POMPOUS ...CONTROVERSIAL... OFFENSIVE ... AND ABUSIVE JERK to hawk their CALMING product!! If █████ is you child... cancel my subscription TODAY!!! Do y...all use child slave labor too????? Tennessee doesn...t need this crap!!* |
| --- |

***Topic 4***

| *█████ The collusion between the Democrats and Russia is appalling! DNC refused to have their computers investigated by the FBI? Hillary and DNC donating millions for the infamous Steele Dossier based on Russian agents! Obama to Putin, after the 2012 election he will have flexibility!*  *------------------------------------------------------------------------------------------------------------------------------------------------------------------------*  *█████ Sorry, I have 6: Harry Styles Louis Tomlinson Niall Horan Liam Payne Zayn Malik One Direction*  *------------------------------------------------------------------------------------------------------------------------------------------------------------------------*  *The █████ app has a sound setting called ...Belfast park. ............... &lt;insert joke here&gt;*  *------------------------------------------------------------------------------------------------------------------------------------------------------------------------*  *█████ .......................... Angry Birds Seasons: █████ Alarm Clock: █████*  *------------------------------------------------------------------------------------------------------------------------------------------------------------------------*  *█████ A pool pump, wet grass, dirt, &amp; some undertone of sound that could be distant waves. Or ships on the horizon.*  *------------------------------------------------------------------------------------------------------------------------------------------------------------------------*  *█████ Roller or balm for wrists and temples. Relaxing candles. Pillow mist. █████. Shakti mat !!*  *------------------------------------------------------------------------------------------------------------------------------------------------------------------------*  *█████ Rocks, pebbles, gravel, hardened clay? .... it...s weird but I loveeeee it.*  *------------------------------------------------------------------------------------------------------------------------------------------------------------------------*  *█████ The imminent threat of societal collapse and environmental disaster :) It fuels me*  *------------------------------------------------------------------------------------------------------------------------------------------------------------------------*  *moisturised, meditated (ty █████ app!), drunk peppermint tea...., but obvs immediately ruined my pro−sleep lifestyle by re−opening twitter....*  *------------------------------------------------------------------------------------------------------------------------------------------------------------------------*  *Las Apps pagas más descargadas para el #iPhone son: 1. Heads Up! 2. Minecraft 3. Afterlight 4. Plague Inc. 5. █████ alarm clock*  *------------------------------------------------------------------------------------------------------------------------------------------------------------------------*  *█████ my first winter in Canada! ......... I am looking forward to: 1. make a snowman ... 2. throw a snowball to my boyfriend ...................... 3. visit winter spa and dip into cold water pond in bikini lol 4. ice skate! ... 5. oops only 1 activity?? too many to mention*  *------------------------------------------------------------------------------------------------------------------------------------------------------------------------*  *........ █████ Yeah but how do I buy Shapeshifters Eden Hazard if I'm catching z's?*  *------------------------------------------------------------------------------------------------------------------------------------------------------------------------*  *█████ █████ YALL R SICK AND TWISTED FOR THIS OMFG*  *------------------------------------------------------------------------------------------------------------------------------------------------------------------------*  *█████ Dave Matthews Busy Phillips (let her have a lullaby too, I miss hers from @BusyTonightTV ) Anna Faris Nick Offerman Emma Thompson Keanu Reeves*  *------------------------------------------------------------------------------------------------------------------------------------------------------------------------*  *█████ Relaxing in the living room on the sofa with the smell of air freshener and bolognese and the sound of my puppy chewing her toys ....*  *------------------------------------------------------------------------------------------------------------------------------------------------------------------------*  *█████ QuickPix, Spotify, Kayak, Orchestra (todos), Calvetica (calendaring), TinyWings, AngryBirds, █████ (alarm)*  *------------------------------------------------------------------------------------------------------------------------------------------------------------------------*  *█████ INSTRUCTIONS UNCLEAR; COMPUTER IS NOW BEATEN WITH HAMMER AND LIT ON FIRE BECAUSE OF SPIN SOUTHWEST RADIO*  *------------------------------------------------------------------------------------------------------------------------------------------------------------------------*  *After mag zoom call ng 11am to 4pm (na may 1 hour lunch break in between), ang sarap pakinggan nung Soundscape sa █████ app na Monastery Drizzle*  *------------------------------------------------------------------------------------------------------------------------------------------------------------------------*  *█████ █████ █████ OMFG I'M DEAD. THE SHOES NOT EVEN ON HIS FEET. HIS HAT THE BEARD HIS OUTFIT. OMG*  *------------------------------------------------------------------------------------------------------------------------------------------------------------------------*  *█████ █████ YEAH I HAVE A QUISTION!! WHY THE HELL DO WE HAVE TO PAY FOR HARRYS DREAM WITH ME* |
| --- |

***Topic 5***

| *█████ ........ CYA. . represents a Clear and Present DANGER for USA and the entire World:HUMANITY!!! Several Points +CYA was created with the purpose to induce terror worldwide,and stArt Ww3/4 CYA +Communications +High Tech +Higher Education +Politics +Pornography +Int.Child Trafficking*  *------------------------------------------------------------------------------------------------------------------------------------------------------------------------*  *█████ Humans were created with a purpose. We developed as all organisms on Earth, serving our purpose within the ecosystem. At some point we asked...why... and we abandoned o purpose to create answers to our questions. We ignore and destroy our ecosystem trying to find our purpose?*  *------------------------------------------------------------------------------------------------------------------------------------------------------------------------*  *MURDERING HEAT DOMES RAINS WINDS SNOW CAP AND TRADE METHANE NATURAL GAS PEAKER PLANTS CARBON CREDITS FRACKING KILLING us WATER VAPOR NITR = 33F. GLOBAL TEMP RISE ACCELERATING YEARLY 220F SLR COLLAPSING ? 2023 ? TRIGGERING DEADLY EARTHQUAKES AND VOLCANOS*  *------------------------------------------------------------------------------------------------------------------------------------------------------------------------*  *So true █████: It is fear alone that limits our potential. Ultimately, anything is possible, potential is limitless █████...*  *------------------------------------------------------------------------------------------------------------------------------------------------------------------------*  *RT █████: It is fear alone that limits our potential. Ultimately, anything is possible, potential is limitless. █████ █████*  *------------------------------------------------------------------------------------------------------------------------------------------------------------------------*  *My Dad &amp; Mom (whom Myself, my Husband, Son &amp; Daughter lived next door to for 20 years) both passed away in 2020. Dad passed 3/14/20 after suffering a heart attack &amp; Mom on 12/26/2020 after a 14 month battle with bone marrow cancer and 8 months after her bone marrow transplant.....*  *------------------------------------------------------------------------------------------------------------------------------------------------------------------------*  *█████ █████ #mindfulness ... the world of a few seconds. With threats. The edge detectors indicates how many distinct persons whom to be an #experience in law, tainted evidence about communicating with higher functions, #MarkovChain.*  *------------------------------------------------------------------------------------------------------------------------------------------------------------------------*  *█████ Sry 1 more point, this pricing makes it a no−brainer not dilemma 4 cash poor, which 4 me is only flaw in a beautiful thing.*  *------------------------------------------------------------------------------------------------------------------------------------------------------------------------*  *█████ Unless you're with an emotional vampire that gaslight you...they make you deny your instincts.*  *------------------------------------------------------------------------------------------------------------------------------------------------------------------------*  *█████ Reminder there is no evidence that these aps are anything more than a scam, a way to make a quick buck for some degenerates. Reminder there is no evidence that these aps are anything more than a scam, a way to make a quickbuck for some degenerates.*  *------------------------------------------------------------------------------------------------------------------------------------------------------------------------*  *█████ █████ █████ I discovered your podcasts from Lex Fridman then wanted to know more about you and went back to your earlier podcasts. Dude I'm complete against Islam but you finding moral ground not to criticize Israel just because palestinans are muslim makes you a complete idiot.*  *------------------------------------------------------------------------------------------------------------------------------------------------------------------------*  *█████ and █████ merging is further proof of the blurring between consumer tech and health tech− and the huge potential*  *------------------------------------------------------------------------------------------------------------------------------------------------------------------------*  *█████ Twitter just informed me the tweets just before this 'could contain offensive material'. Their standards must be beyond angelic if anything here is offensive! ....*  *------------------------------------------------------------------------------------------------------------------------------------------------------------------------*  *█████ The road rage question was spot on. He didn...t answer it. Isn...t it true that all emotions triggered from an experience &amp; directed towards an object? I would assume that life as a ...non−subject... in a world with ...no objects... would be emotionless.*  *------------------------------------------------------------------------------------------------------------------------------------------------------------------------*  *█████ Our mom has Narcissistic Personality Disorder, RESEARCH; Seek █████, █████, █████, Jocko Willink, Eckhart Tolle; Emigrate from th Philippines &amp; head for San Diego Cali.; Buy Apple Stock &amp; take up Electrical Engineering; avoid serious relationships!*  *------------------------------------------------------------------------------------------------------------------------------------------------------------------------*  *Lima app/game berbayar terbaik di App Store: 1. Heads up! 2. Minecraft PE 3. Afterlight 4. Plague 5. █████ alarm*  *------------------------------------------------------------------------------------------------------------------------------------------------------------------------*  *█████ I agree with the sentiment, but can't ignore that apostrophe! Considered acknowledging and accepting but it's impossible ;)*  *------------------------------------------------------------------------------------------------------------------------------------------------------------------------*  *█████ █████ Excellent! Have just started the cancer pack...to help me cope with #Lupus. I just substitute the word "Lupus" for "cancer".*  *------------------------------------------------------------------------------------------------------------------------------------------------------------------------*  *█████ My wood burning sauna in a 100 year old wooden house. ....*  *------------------------------------------------------------------------------------------------------------------------------------------------------------------------*  *█████ HOW COULD YOU END THE PODCAST IM HIGHLY OFFENDED, HURT, DISTRAUGHT, AND A LITTLE HUNGRY. BUT MOSTLY HURT* |
| --- |

***Topic 6***

| *█████ my faves−−Instagram, Pepperplate (managing recipes), Yelp, Target, a CTA tracker (RedEye or myTransit) & █████ (alarm, $.99)*  *------------------------------------------------------------------------------------------------------------------------------------------------------------------------*  *Trying out the █████ app for jet lag. It monitors sleep patterns and activates the morning alarm during the lightest sleep phase.*  *------------------------------------------------------------------------------------------------------------------------------------------------------------------------*  *█████ █████ █████ The █████ App for iOS is a game changer. Wakes you up at the end of a cycle so as to not startle you awake from REM sleep. ........*  *------------------------------------------------------------------------------------------------------------------------------------------------------------------------*  *█████ So whichever applies start there!!! If you...re waking up to an alarm and feeling exhausted and groggy then switch to █████ app. Its amazing. It wakes you up in your lightest stage of sleep so waking feels natural and you...re not woken up in the dead of sleep.*  *------------------------------------------------------------------------------------------------------------------------------------------------------------------------*  *█████ A nap this afternoon − between my neighbours... flashing Christmas lights and a dog with an upset stomach I am not feeling daisy−fresh!*  *------------------------------------------------------------------------------------------------------------------------------------------------------------------------*  *i might be enduring acid reflux, a really bad bpd episode, and 24% quality on my █████ app but cha hakyeon looked me dead in my eyes and spoke very gently and smiled at me on saturday so i'm gonna. .... Live With Monday.*  *------------------------------------------------------------------------------------------------------------------------------------------------------------------------*  *█████ $2 ... █████... App on my iPhone...It analyzes sleep quality and REM cycles...and most importantly, wakes me up within a general time−frame I choose when I...m m awake, rather than an abrupt alarm in deep sleep.*  *------------------------------------------------------------------------------------------------------------------------------------------------------------------------*  *Randomly drank a Guinness, a bud light seltzer, and a tall boy IPA tonight so while my body figures out what to do with that information I...m gonna throw on the █████ app and go to sleep*  *------------------------------------------------------------------------------------------------------------------------------------------------------------------------*  *█████ █████ part ..................... and modify the chemicals that allows that. I guess my comment is about how the chemicals are modified by mindfullness and our feelings are not the inexorable result of neurotransmiters activity. Any insight on this topic?*  *------------------------------------------------------------------------------------------------------------------------------------------------------------------------*  *█████ Simon...s sleep casts and █████ sessions − I find his voice and calamity hypnotising ........*  *------------------------------------------------------------------------------------------------------------------------------------------------------------------------*  *█████ Looking forward to Christmas Din Din with the Fam!*  *------------------------------------------------------------------------------------------------------------------------------------------------------------------------*  *█████ █████: alarm clock and sleep tracker. Its unique design only wakes you up while you...re in your light sleep phase.*  *------------------------------------------------------------------------------------------------------------------------------------------------------------------------*  *█████ Dang! My █████ app is collecting dust. Digital dust ...*  *------------------------------------------------------------------------------------------------------------------------------------------------------------------------*  *Ok party people, I'm totally addicted and fascinated by the █████ app! Always knew I sucked at sleeping but WOW my graphs r shocking!*  *------------------------------------------------------------------------------------------------------------------------------------------------------------------------*  *█████ Aaaahhh this happend to me this morning − soooooo frustrating!*  *------------------------------------------------------------------------------------------------------------------------------------------------------------------------*  *█████ What u need is the █████ App for lphone. Measures your sleep pattern & wakes you up in light sleep zone, no jolt to awake*  *------------------------------------------------------------------------------------------------------------------------------------------------------------------------*  *According to the █████ app I get on average 7hrs 9mins of sleep, I twitch a lot and my dreams are of the perverted nature. According to my █████ app, my sleep quality was 24% last night. Looking forward to the headache this afternoon.*  *------------------------------------------------------------------------------------------------------------------------------------------------------------------------*  *█████ █████ █████ The █████ App for iOS is a game changer. Wakes you up at the end of a cycle so as to not startle you awake from REM sleep. ........*  *------------------------------------------------------------------------------------------------------------------------------------------------------------------------*  *█████ Hard egg nog with an xtra shot o...scotch* |
| --- |

***Topic 7***

| *█████ And with those who say:"Lo! we are Christians," We made a covenant, but they forgot a part of that whereof they were admonished. Therefore We have stirred up enmity and hatred among them till the Day of Resurrection, when Allah will inform them of their handiwork. UNITE WORKERS*  *------------------------------------------------------------------------------------------------------------------------------------------------------------------------*  *█████ Vulnerability is strength. You're not strong because you act tough and loud to hide your insecurity. You're strong if you can admit and accept those insecurities and act in spite of them. Acceptance of insecurity requires the vulnerability to admit they're part of you.*  *------------------------------------------------------------------------------------------------------------------------------------------------------------------------*  *█████ Rainday Antiques &amp; Slow Train (kudos to Simon) Moon buggy (tx Yohannes!)*  *------------------------------------------------------------------------------------------------------------------------------------------------------------------------*  *█████ HOW TO HACK YOUR SEROTONIN ...THE REGULATORY HORMONE... 1) Physical Exercise 2) Meditation 3) Vitamin D + K 4) Sunlight 5) Eat Nuts, Oats, Seeds, Pineapple And S*  *------------------------------------------------------------------------------------------------------------------------------------------------------------------------*  *█████...s mvp was meditation meetups. Kinda sweaty startup−esque. Tech is secondary!*  *------------------------------------------------------------------------------------------------------------------------------------------------------------------------*  *█████ Le Bron is annoying &amp; conceited. Why would I boost his already inflated ego?*  *------------------------------------------------------------------------------------------------------------------------------------------------------------------------*  *█████ █████ Moon Buggy or Rainy Day Antiques... for the ZZZZs....*  *------------------------------------------------------------------------------------------------------------------------------------------------------------------------*  *█████ SEMANTIC SATIATION ... A Word/Phrase Can Temporarily Lose It...s Meaning Because Of Repetition ... Due To A Trigger Of Neural Pattern Associated With Words/Phrases Inhe Meaning Then Decreasing Neural Activity ... The Quality Of Our Words Is Far More Important Than The Quantity*  *------------------------------------------------------------------------------------------------------------------------------------------------------------------------*  *█████ Falling snow is my favorite. Reminds me of my time in Lake Tahoe &amp; also a favorite lyric of mine ...a freshly fallen silen shroud of snow...*  *------------------------------------------------------------------------------------------------------------------------------------------------------------------------*  *Forgot my darn headphones &amp; really want to try out the █████ app I just downloaded. Can't be as offensive as East 17 on my ghetto blaster circa 1996 in Tenerife. Can it?*  *------------------------------------------------------------------------------------------------------------------------------------------------------------------------*  *█████ WAIT SO IS HE ACTUALLY READIING US A STORY AND KTS NOT JSUT HUS MUSIC SLOWED FOWN AKSKDODP*  *------------------------------------------------------------------------------------------------------------------------------------------------------------------------*  *#toptips for #study; Don't bite off more than you can chew Don't fall behind █████ app Don't fall behind #exercise Dont fall behind*  *------------------------------------------------------------------------------------------------------------------------------------------------------------------------*  *█████ IF I HAVE A BAD DAY I HAV SUM CANS OF STELLA AND PLAY ON LADBROOKS CASINO !!!!!!!!!*  *------------------------------------------------------------------------------------------------------------------------------------------------------------------------*  *█████ My MENLA Kundalini retreat in the Catskills &amp; forest, along with a spa facial in the late morning! ....*  *------------------------------------------------------------------------------------------------------------------------------------------------------------------------*  *█████ Any vague ETA on Psychedelic track? And Hypnosis tack?*  *------------------------------------------------------------------------------------------------------------------------------------------------------------------------*  *█████ █████ #mindfulness is the world in a few pharmacological shortcuts exist and body as far more to be couched in is terrifying social policy that we can do you turn fleeting memory loss. #MarkovChain.*  *------------------------------------------------------------------------------------------------------------------------------------------------------------------------*  *█████ meditation wind downs, rain sounds, doze music on █████ app, series of yoga poses before bed, light reading, (i have a book literally called sleep by lisa varadi) cbd, kava kava, smother urself w lavender essential oil &amp; put it in a diffuser if you have one!*  *------------------------------------------------------------------------------------------------------------------------------------------------------------------------*  *█████ Midnight Launderette, Rainy Day Antique, Slow Train. Sandy Cove*  *------------------------------------------------------------------------------------------------------------------------------------------------------------------------*  *█████ Morning meditation, morning yoga, coffee with wife, skateboarding, float tank, grill out with family and sunset walk evening meditation and donuts*  *------------------------------------------------------------------------------------------------------------------------------------------------------------------------*  *█████ "We can &amp; we will steal Seerut K Chawla's words"* |
| --- |

***Topic 8***

| *█████ How about specific support for all the young MUSLIMS affected by the constant barrage of Islamophobic terror? As a 50+ Aussie woman I'm outraged sad, everything else, imagine the kids!*  *------------------------------------------------------------------------------------------------------------------------------------------------------------------------*  *█████ Inhale&gt; Your favorite scent ex: lavender, orange blossom, freesia, lily, chocolate chip cookies etc. Exhale&gt; Negativity, stress, anxiety*  *------------------------------------------------------------------------------------------------------------------------------------------------------------------------*  *█████ app coming in clutch right now to drown out person at library using dry erase markers ...screech screech screech. ...................... thank god for voitures sous la pluie! #newsubscriber #cybermonday*  *------------------------------------------------------------------------------------------------------------------------------------------------------------------------*  *█████ IM SCREAMINGGG PLEASEE FUCKING YESSS BBYYY*  *------------------------------------------------------------------------------------------------------------------------------------------------------------------------*  *█████ Sorry one more question.Says guided and unguided on the singles pack,not too sure what that means,&amp; if itjust relates to the circles marked?*  *------------------------------------------------------------------------------------------------------------------------------------------------------------------------*  *█████ Right... Meditation use to remove the roots of past trauma... Crying is to remove roots of recent trauma.. Both are doing same to remove trauma.....*  *------------------------------------------------------------------------------------------------------------------------------------------------------------------------*  *█████ █████ █████ fuck FUCK FUCK FUCK FUCK*  *------------------------------------------------------------------------------------------------------------------------------------------------------------------------*  *█████ And sadly not your victories ;) and success ;) trouble is we so readily define ourselves by our success and even encouraged to do so by society. At the same time, we pretend we can talk ourselves out of our identification with our miseries. We are pretty sad beings ....*  *------------------------------------------------------------------------------------------------------------------------------------------------------------------------*  *If the █████ app and the █████ app were to merge, it would be the uber−super−perfect alarm app! Seriously, folks..hook up, please!*  *------------------------------------------------------------------------------------------------------------------------------------------------------------------------*  *█████ WJAT THE FUCK IS HAPENEDK NDCNF*  *------------------------------------------------------------------------------------------------------------------------------------------------------------------------*  *█████ have you read your terms of use recently? Forced arbitration, forced opt−out of everything, horrible formatting of both ToS and privacy agreement. 13.12 f is a joke .... shame, was really looking forward to trying your app out*  *------------------------------------------------------------------------------------------------------------------------------------------------------------------------*  *█████ ooops sorry we have broken your app*  *------------------------------------------------------------------------------------------------------------------------------------------------------------------------*  *█████ exhausted,can...t focus,stressed and pretty anxious tdy.not the bad day that...s for sure ....*  *------------------------------------------------------------------------------------------------------------------------------------------------------------------------*  *█████ have you problems with the Phillips Hue 2nd Generation? (The square box). I can't linked with your App.*  *------------------------------------------------------------------------------------------------------------------------------------------------------------------------*  *Weirdly impressed by the vertical format animations embedded in the █████ app.*  *------------------------------------------------------------------------------------------------------------------------------------------------------------------------*  *█████ Fucking traumatised watching Euro 2020....*  *------------------------------------------------------------------------------------------------------------------------------------------------------------------------*  *My █████ app says: "Make sure you're somewhere where you won't be disturbed." My #cat says: "Mrrrp. Meow." *Headbump*. ....*  *------------------------------------------------------------------------------------------------------------------------------------------------------------------------*  *█████ Testing your app &amp; I...m confounded by how LOUD the default volume is on [music, SFX, stories,etc] ... with no apparent way to adjust the volume, in app. If the answer = turn down iPhone volume, then alarm volume is compromised. Unusable. Am I missing something?*  *------------------------------------------------------------------------------------------------------------------------------------------------------------------------*  *█████ █████ █████ SHUT THR FUCK UPPP*  *------------------------------------------------------------------------------------------------------------------------------------------------------------------------*  *█████ How do I escalate a fault on my app which I raised on 5 Aug, is with the engineers but "Sadly, we don't currently have an estimate for when the issue might be fully resolved"* |
| --- |

***Topic 9***

| *KB Hotlist − Wizards 17 in a row, Left Hand Milk Stout, Raku Ramen,Tony Kornheiser pod, imgur,Headspace App, Catastrophe, @trumpdraws , naps*  *------------------------------------------------------------------------------------------------------------------------------------------------------------------------*  *█████ Lmfao. Ben will finance her biting you or brandy will help me perform a penile fasciotomy.*  *------------------------------------------------------------------------------------------------------------------------------------------------------------------------*  *█████ █████ In Iran, people are killed . #Mahsa_Amini and #Nika_Shah_Karmi are killed. because of the hijab because of their hair, the Islamic revolution guards corps is killing people, help*  *------------------------------------------------------------------------------------------------------------------------------------------------------------------------*  *█████ █████ It's a great sleep story. But, there's a factual error. Jews weren't allowed to return to England till after the 1650s. The oldest synagogue in Britain, Bevis Marks in London, was built in 1701. The narrator's claim that, in 1602, Edward would have seen synagogues is inaccurate*  *------------------------------------------------------------------------------------------------------------------------------------------------------------------------*  *my █████ app is supposed to record me snoring but most of the recordings are of ringo snoring or him hissing at bowie*  *------------------------------------------------------------------------------------------------------------------------------------------------------------------------*  *█████ "Eureka Street" by Robert Mcliam Wilson or "Dark lies the island" Kevin Barry*  *------------------------------------------------------------------------------------------------------------------------------------------------------------------------*  *█████ Aloe Vera gel for the sunburn, █████ app for the deadline stress. Sorted ....*  *------------------------------------------------------------------------------------------------------------------------------------------------------------------------*  *█████ █████ You're spot on, Gary. Everything is now hidden in a drop down. Right now, for me, clicking any section in that drop down provides me with an empty scene. What's worse is I've downloaded packs offline and when im offline finding them is a huge hassle*  *------------------------------------------------------------------------------------------------------------------------------------------------------------------------*  *█████ YOUR JOKING THIS IS A JOKE OH MY GOD OH NY GOD OH NY GSIAVQHQIQNQJJQBWW*  *------------------------------------------------------------------------------------------------------------------------------------------------------------------------*  *█████ Roger apron, (Mr/Monk nahascene. (R.i.p) Billows are purple, sun picks up the seeds.....*  *------------------------------------------------------------------------------------------------------------------------------------------------------------------------*  *█████ █████ This is craziness? It's actually abhorrent that you don't support his actual message: "And the truth is that no pro−life person on earth would kill baby Hitler, because baby Hitler wasn't Hitler, adult Hitler was Hitler. Baby Hitler was a baby," he continued.*  *------------------------------------------------------------------------------------------------------------------------------------------------------------------------*  *█████ I blame █████ Billy Joel Channel, but give me "Shades of Grey", "Zanzibar", or "Until the Night".*  *------------------------------------------------------------------------------------------------------------------------------------------------------------------------*  *Stressed that my █████ app is malfunctioning when trying to download a stress pack to help with stress. Talk about #meta*  *------------------------------------------------------------------------------------------------------------------------------------------------------------------------*  *█████ I don't believe in this bullcrap satan worship garbage*  *------------------------------------------------------------------------------------------------------------------------------------------------------------------------*  *█████ █████ You do not align with the idea that it is wrong to kill babies? Bold stance to take. I can only conclude that Calm believes baby murder is acceptable.*  *------------------------------------------------------------------------------------------------------------------------------------------------------------------------*  *█████ you are okay will killing babies? If you pull sponsorship from █████, that is your stance. Period.*  *------------------------------------------------------------------------------------------------------------------------------------------------------------------------*  *█████ The creak of bicycle spokes while riding a bike*  *------------------------------------------------------------------------------------------------------------------------------------------------------------------------*  *█████ █████ Okay I'm confused. So you're saying that you would kill baby Hitler? That's what I get from you pulling your adverts from the show. Ben is Jewish and he is saying that even he wouldn't kill baby Hitler. Because it's a baby. Come on man*  *------------------------------------------------------------------------------------------------------------------------------------------------------------------------*  *█████ █████ █████ − visit a herbal medicine practitioner, many herbs to help anxiety and coupled with a holistic approach..*  *------------------------------------------------------------------------------------------------------------------------------------------------------------------------*  *OH &amp; I are doing the █████ app. You know to help us with stress, being more mindful, etc. Tonight...s episode was accompanied by the sounds of the hamster trying to break out of his cage, 6yr old...s toy tiger roaring &amp; 3yr old having coughing fit. I...m the Dalai Lama now.* |
| --- |

***Topic 10***

| *█████ Currently ...Fooled Around and Fell In Love by Miranda Lambert with Elle King , Maren Morris, Ashley McBryde, Tenille Townes, and Caylee Hammack.*  *------------------------------------------------------------------------------------------------------------------------------------------------------------------------*  *█████ My brain is made up of 12−15 tone deaf and highly caffeinated monkeys all equipped with miscellaneous percussive and woodwind instruments.*  *------------------------------------------------------------------------------------------------------------------------------------------------------------------------*  *█████ █████ He...s a fraud and owned by China. █████ most overrated player in nba history.*  *------------------------------------------------------------------------------------------------------------------------------------------------------------------------*  *█████ █████ If this were true − every political division would cease henceforth! Take a look at politics − it dictates the irrationalities of the status quo : by dominance &amp; deception: it...s financial markets for inequality to proliferate!*  *------------------------------------------------------------------------------------------------------------------------------------------------------------------------*  *Join us live on LinkedIn and YouTube on Thursday, May 26 at 1:00 p.m. PT / 4:00 p.m. ET for this important conversation moderated by Dr. █████, licensed psychologist, trauma expert and Chief Social Impact and Diversity Officer at █████ Health.*  *------------------------------------------------------------------------------------------------------------------------------------------------------------------------*  *█████ █████ Sir you recently said that Biden was bumbling, incoherent, etc. His recent speech in Cleveland belies that. It's true: Biden rarely says something nonsensical, but most Republicans rarely say something sensical. Ie "YOU'RE just wrong." This only helps the GOP.*  *------------------------------------------------------------------------------------------------------------------------------------------------------------------------*  *█████ Restaurant takeaway out. Made cheese soufflé, lie in with furry snuggles..*  *------------------------------------------------------------------------------------------------------------------------------------------------------------------------*  *█████ I quote tweeted one of █████ maniacal rants about █████ saying " █████ has Trump derangement syndrome. █████ has █████ derangement syndrome." And █████ blocked this account! I've got 7 followers ....*  *------------------------------------------------------------------------------------------------------------------------------------------------------------------------*  *█████ Yeah, you don't know that and the one−dimensional cult of positivity this statement implies is dangerous and harmful.*  *------------------------------------------------------------------------------------------------------------------------------------------------------------------------*  *█████ █████ █████ █████ Every considered doing a conversation with █████? You are both rigorously scientific and converging on the same problem albeit with slightly different terms. Why haven't you spoke to each other? Could be enlightening.*  *------------------------------------------------------------------------------------------------------------------------------------------------------------------------*  *█████ and █████, I couldn...t focus during my mediation after you said ...Shake it off,... is a Mariah Carey song, it...s a Taylor Swift song! Sooo distracting! █████*  *------------------------------------------------------------------------------------------------------------------------------------------------------------------------*  *█████ Pulling weeds along our fence line. Hubby has a weed eater, but I love playing in the dirt!*  *------------------------------------------------------------------------------------------------------------------------------------------------------------------------*  *█████ Stop bullying. Stop with the BS that gets framed as ...office politics.... Stop having toxic work colleagues but framing it as ...oh it...s just their character ....... Stop treating people differently because of their skin colour but framing it as ...you need to be more approachable....*  *------------------------------------------------------------------------------------------------------------------------------------------------------------------------*  *█████ Folding laundry: smoothing all those wrinkles out and matching every corner. ....*  *------------------------------------------------------------------------------------------------------------------------------------------------------------------------*  *█████ Yes, moods are contagious and such an improper ant component of our daily interactions as humans! Thanks!*  *------------------------------------------------------------------------------------------------------------------------------------------------------------------------*  *█████ Interesting and important topic. Passive aggressive undercurrent in the discussion, unfortunately.*  *------------------------------------------------------------------------------------------------------------------------------------------------------------------------*  *█████ Are liberals intentionally distorting █████...s message? Yes 100% (598 Votes) No 0% (0 Votes) BIG MISTAKE BIG*  *------------------------------------------------------------------------------------------------------------------------------------------------------------------------*  *█████ Knock − knock! Who's there? Orange! Orange who? Orange you glad your alive?*  *------------------------------------------------------------------------------------------------------------------------------------------------------------------------*  *█████ █████ To anyone who needs this: Every failure, every discouragement, every naysayer − don...t let them stop you from succeeding. Learn from every failure, and seek to prove every doubter wrong. Defy adversity. Have a lovely day!*  *------------------------------------------------------------------------------------------------------------------------------------------------------------------------*  *█████ Sell it to Teflon Dons supporters. They need to reflect quietly on the damage he his doing and the division he is creating.* |
| --- |
